# Author Correction: Electron diffraction of deeply supercooled water in no man’s land

**DOI:** 10.1038/s41467-025-57398-1

**Published:** 2025-03-03

**Authors:** Constantin R. Krüger, Nathan J. Mowry, Gabriele Bongiovanni, Marcel Drabbels, Ulrich J. Lorenz

**Affiliations:** Ecole Polytechnique Fédérale de Lausanne (EPFL), Laboratory of Molecular Nanodynamics, CH-1015 Lausanne, Switzerland

Correction to: *Nature Communications* 10.1038/s41467-023-38520-7, published online 17 May 2023

In the version of the article initially published, Supplementary Note J on determination of the location of the hypothetical Widom line was not included in the Supplementary information, while in the second to last paragraph of the article, in the text now reading “Taking into account the temperature evolution of the high- and of the low-temperature liquid, one can estimate the location of the hypothetical Widom line to occur at 234 ± 1 K using the procedure suggested in Ref. 24 (see Supplementary Note J),” 234 ± 1 K appeared as “238 ± 1 K” and the callout to Supplementary Note J was missing. The Supplementary information and HTML and PDF versions of the article are now updated.

## Supplementary information

Updated Supplementary Information

